# Thrombocytopenia in neonates and the risk of intraventricular hemorrhage: a retrospective cohort study

**DOI:** 10.1186/1471-2431-11-16

**Published:** 2011-02-11

**Authors:** Jeannette S von Lindern, Tjitske van den Bruele, Enrico Lopriore, Frans J Walther

**Affiliations:** 1 Division of Neonatology J6-S, Department of Pediatrics, Leiden University Medical Center, PO Box 9600, 2300 RC Leiden, The Netherlands; 2 Department of Pediatrics, Groene Hart Hospital, Gouda, The Netherlands

## Abstract

**Background:**

The overall prevalence of thrombocytopenia in neonates admitted to neonatal intensive care units ranges from 22 to 35%. There are only a few small studies that outline the relationship between the severity of thrombocytopenia and the risk of bleeding. This makes it difficult to form an evidence-based threshold for platelet transfusions in neonatal patients. The aim of this study was to determine the prevalence of thrombocytopenia in a tertiary neonatal intensive care unit and to study the relation between thrombocytopenia and the risk of intraventricular hemorrhage (IVH).

**Methods:**

We performed a retrospective cohort study of all patients with thrombocytopenia admitted to our neonatal tertiary care nursery between January 2006 and December 2008. Patients were divided into 4 groups according to the severity of thrombocytopenia: mild (100-149 × 10^9^/L), moderate (50-99 × 10^9^/L), severe (30-49 × 10^9^/L) or very severe (< 30 × 10^9^/L). The primary outcome was IVH ≥ grade 2. Pearson's chi-squared and Fischer's exact tests were used for categorical data. ANOVA, logistic regression analysis and multivariate linear regression were used for comparisons between groups and for confounding factors.

**Results:**

The prevalence of thrombocytopenia was 27% (422/1569). Risk of IVH ≥ grade 2 was 12% (48/411) in neonates with versus 5% (40/844) in neonates without thrombocytopenia (p < 0.01). After multivariate linear regression analysis, risk of IVH ≥ grade 2 in the subgroups of thrombocytopenic infants was not significantly different (p = 0.3).

After logistic regression analysis the difference in mortality rate in neonates with and without thrombocytopenia was not significant (p = 0.4). Similarly, we found no difference in mortality rate in the subgroups of neonates with thrombocytopenia (p = 0.7).

**Conclusion:**

Although IVH ≥ grade 2 occurs more often in neonates with thrombocytopenia, this relation is independent of the severity of thrombocytopenia. Prospective studies should be conducted to assess the true risk of hemorrhage depending on underlying conditions. Randomized controlled trials are urgently needed to determine a safe lower threshold for platelet transfusions.

## Background

The overall prevalence of thrombocytopenia in neonates ranges from 1 to 5%[1-3] and is reported to be much higher in neonates admitted to neonatal intensive care units, ranging from 22 to 35%[1-6]. From 22 weeks' gestation onwards, the platelet count reaches and maintains a level above 150 × 10^9^/L, thereby defining thrombocytopenia in the newborn as a platelet count below 150 × 10^9^/L[1,2]. Many neonatal and maternal conditions are associated with thrombocytopenia, of which septicemia and prematurity are the most common[2,6]. In thrombocytopenia the major concern is an increased risk of bleeding.

In 1882 Bizzozzero was the first to describe the role of platelets in coagulation and thrombosis[7]. Since then, only a few small studies investigating the relationship between the severity of thrombocytopenia and the risk of bleeding in newborns have been reported [4,8,9]. Likewise, the number of clinical trials examining thrombocytopenia and the effects on bleeding in adults is limited[10-13]. The lack of studies makes it difficult to form an evidence-based threshold for platelet transfusions in neonatal patients.

The aim of this study was to analyze and describe all cases with thrombocytopenia admitted to our neonatal nursery during a 3-year period and study a possible relationship between the risk of intraventricular hemorrhage (IVH) and the severity of thrombocytopenia. We studied the prevalence and risk factors of thrombocytopenia in relation to the risk of IVH and mortality.

## Methods

### Patients

We retrospectively collected data from all neonates admitted between January 2006 and December 2008 to the neonatal department of the Leiden University Medical Center, a tertiary neonatal care center in The Netherlands. In the Netherlands no ethical approval is required for this type of research as no new intervention or treatment is studied. Nor is any randomization needed. All collected data was anonymized. We identified all thrombocytopenic newborns by extracting data from our dedicated patient-database, medical files, laboratory system and electronic blood banking records. We excluded neonates with only one platelet count measurement below 150 × 10^9^/l. We considered these isolated counts as clotted samples, platelet clumping, laboratory error or one-time only measurements with immediate normalization. Thrombocytopenia was defined as a platelet count below 150 × 10^9^/L. The included neonates with thrombocytopenia were divided into 4 groups, based on their lowest platelet count during their stay in our unit, and classified as mild (platelet count 100-149 × 10^9^/L), moderate (platelet count 50-99 × 10^9^/L), severe (platelet count 30-49 × 10^9^/L) or very severe (platelet count < 30 × 10^9^/L), according to standard classification[1,2,6,14-17].

We recorded the presence of IVH detected by cranial ultrasound and classified according to Volpe[18]. IVH grade 2, grade 3 or grade 4 (i.e. periventricular hemorrhagic infarction (PVHI)) were recorded.

Cranial ultrasounds were performed according to local protocol depending on gestational age and degree of illness.

Data for demographic as well as clinical conditions of all infants were collected, including gender, gestational age at birth, birth weight, small for gestational age, chromosomal disorders, perinatal asphyxia, necrotizing enterocolitis, sepsis/meningitis, hemorrhage, thrombosis, central catheters, polycythemia, rhesus hemolytic disease, exchange transfusion, neonatal allo-immune thrombocytopenia and the number of blood product transfusions (platelets, erythrocytes, fresh frozen plasma). Small for gestational age was defined as a birth weight < 3^rd ^percentile for the corresponding gestational age[19]. Chromosomal disorders were defined as congenital anomalies related to thrombocytopenia, such as trisomy 18 and 21. Perinatal asphyxia was defined as a five minute Apgar score < 5, a decelerative heart rate on a cardiotocogram and/or an arterial umbilical cord pH below 7.0. Hypotension was defined as a mean blood pressure below the 3^rd ^percentile for gestational age and requiring inotropic support. Sepsis was defined as a positive blood culture in a neonate with clinical signs of infection. Necrotizing enterocolitis was scored based on Bell staging criteria[20]. Polycythemia was defined as a venous hematocrit ≥ 65% in symptomatic infants or ≥ 70% with or without symptoms. A thrombus could be catheter related, in a major blood vessel or intracardial, detected with ultrasound.

The primary outcome measure was IVH ≥ grade 2. The secondary outcomes were total number of platelet transfusions and mortality.

In our hospital a platelet transfusion for neonates is a concentrated single donor product in plasma and is leukocyte depleted. The dose is a median of 20 × 10^9 ^platelets per kg. The product is irradiated with 25 Gy for all infants with a gestational age below 32 weeks and/or a birth weight below 1500 grams and/or for neonates that previously underwent an intra-uterine transfusion. Guidelines for platelet transfusions in our department were as follows: 1) platelet count < 30 × 10^9^/L and stable, 2) platelet count < 50 × 10^9^/L and unstable, and/or birth weight < 1000 g, and/or previous major bleeding, and/or after exchange transfusion, and/or before planned surgery and/or rapid decrease of platelets, or 3) platelet count < 100 × 10^9^/L in neonates with active bleeding and/or at start of exchange transfusion[17].

### Statistics

Data analyses were performed using Statistical Package for Social Sciences (SPSS), version 16.0 (SPSS, Inc., Chicago, Illinois, USA). For every separate variable the Pearson's chi-squared test was used. If the chi-squared-test could not be used (frequency of an event was < 5) the Fisher's exact test was used. Comparisons between group means were analyzed using the one way ANOVA test (with a 95% confidence-interval). Logistic regression was performed to evaluate the confounders between the infants with and without thrombocytopenia. Factors considered potential confounders were variables with a significant difference in thrombocytopenia. Multivariate linear regression was used to compare for confounders in the subgroups of thrombocytopenic neonates, because of the small number in some of the subgroups of thrombocytopenic neonates. A p-value smaller than 0.05 was considered to be significant.

## Results

### Total patient population

**Figure 1:**
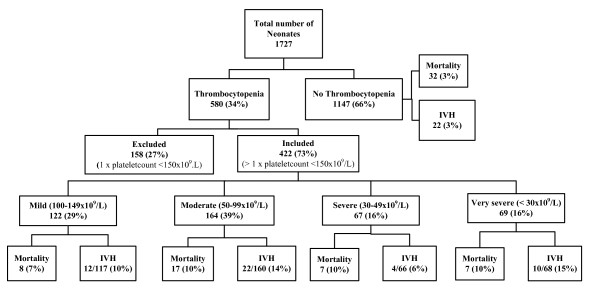
**Flow-chart of all in- and exclusions of the cohort**.

A total of 1727 neonates were admitted to our neonatal nursery during the 3-year study period. Thrombocytopenia was detected in 580 neonates, of which 158 were excluded because of only one platelet count below 150 × 10^9^/L. The prevalence of thrombocytopenia was 27% (422/1569). Neonates with thrombocytopenia were divided into four groups according to their lowest platelet count; 122 (29%) mild, 164 (39%) moderate, 67 (16%) severe and 69 (16%) with very severe thrombocytopenia. The distribution of included and excluded neonates is shown in Figure 1.

An overview of the baseline characteristics of all included neonates with (n = 422) and without (n = 1147) thrombocytopenia is presented in Table 1. Except for gender, single or multiple births and chromosomal disorders, every characteristic was significantly different.

**Table 1:** Patient characteristics of neonates with and without thrombocytopenia.

	Neonates without thrombocytopenia (N = 1147)	Neonates with thrombocytopenia (N = 422)	P-value
Female gender, n (%)	513 (45)	179 (42)	0.41

GA at birth (weeks), mean ± SD	35 ± 4.2	32.5 ± 4.8	<0.01

Birth weight (gram), mean ± SD	2537 ± 968	1864 ± 1067	<0.01

SGA, n (%)	44 (4)	62 (15)	<0.01

Multiple births (single), n (%)	270 (24)	100 (24)	0.96

**Postnatal condition**			
Asphyxia, n (%)	42 (4)	46 (11)	<0.01

Hypotension, n (%)	68 (6)	123 (29)	<0.01

Sepsis, n (%)	77 (7)	129 (30)	<0.01

NEC, n (%)	9 (1)	10 (2)	0.01

Central catheter, n (%)	403 (35)	346 (82)	<0.01

Thrombus, n (%)	6 (1)	14 (3)	<0.01

NAITP, n (%)	1 (0)	14 (3)	<0.01

Polycythemia, n (%)	32 (3)	26 (6)	<0.01

RHD, n (%)	61 (5)	40 (10)	<0.01

Exchange transfusion, n (%)	1 (0)	14 (3)	<0.01

Chromosomal disorders, n (%)	9 (1)	7 (2)	0.15

GA = gestational age, SGA = small for gestational age, NEC = necrotizing enterocolitis, NAITP = Neonatal alloimmune thrombocytopenia, RHD = Rhesus Hemolytic Disease.

### Primary and secondary outcome in total patient population

Cranial ultrasound was performed in 97% (411/422) of neonates with thrombocytopenia and in 74% (844/1147) of infants without thrombocytopenia. The rate of IVH ≥ grade 2 in neonates with and without thrombocytopenia was 12% (48/411) and 5% (40/844), respectively (p < 0.01). After multiple regression analysis, with all significantly different variables, the correlation between IVH and thrombocytopenia was still statistically significant (p = 0.045); gestational age remained an independent significant risk factor for IVH (p < 0.01).

Mortality rate in neonates with and without thrombocytopenia, respectively 9% (39/422) vs. 3% (32/1147), was not significantly different after multiple regression analysis (p = 0.4).

### Thrombocytopenic patient population

Thrombocytopenia was detected at a mean of 2 days after birth (range 0-56 days). In the group of thrombocytopenic neonates (n = 422), 27 died before thrombocytopenia had resolved and in 32 neonates laboratory testing was discontinued before a platelet count above 150 × 10^9^/L was recorded during follow-up. In these 32 infants platelet counts were not measured because of already increasing platelet counts with a value above 120 × 10^9^/L. In the remaining 363 neonates, the mean duration of thrombocytopenia was 9 days (range 0-112 days). We found a significant positive correlation between severity of thrombocytopenia and the time to recovery. Duration of thrombocytopenia in the mild, moderate, severe and very severe group was 5, 8, 10 and 16 days, respectively (p < 0.01).

Mean gestational age at birth was 32.5 (range 24 to 42) weeks. Of all thrombocytopenic neonates, 75% (316/422) were preterm (<37 weeks). Patient characteristics divided into subgroups according to severity of thrombocytopenia are shown in Table 2. After linear regression analysis, severity of thrombocytopenia remained associated with sepsis (p < 0.01) and thrombi (p < 0.01).

**Table 2:** Patient characteristics of thrombocytopenic neonates, divided into subgroups by severity.

	Mild **(100-149 × 10**^**9**^**/L)** (N = 122)	Moderate **(50-99 × 10**^**9**^**/L)** (N = 164)	Severe **(30-49 × 10**^**9**^**/L)** (N = 67)	Very severe **(<30 × 10**^**9**^**/L)** (N = 69)	P-value
	
Gender (female), n (%)	51 (42)	66 (40)	32 (48)	30 (43)	0.77
GA (weeks), mean ± SD	32.8 ± 4.7	32.1 ± 4.7	32.5 ± 4.7	32.9 ± 5.0	0.56

Weight (gram), mean ± SD	2004 ± 1172	1715 ± 984	1854 ± 1058	1979 ± 1046	0.11

SGA, n (%)	13 (11)	29 (18)	12 (18)	8 (12)	0.28

**Postnatal condition**					

Asphyxia, n (%)	11 (9)	14 (9)	9 (13)	12 (17)	0.19

Hypotension, n (%)	27 (22)	46 (28)	28 (42)	22 (32)	0.04

Sepsis, n (%)	26 (21)	43 (26)	25 (37)	35 (51)	<0.01

NEC, n (%)	1 (1)	4 (2)	3 (4)	2 (3)	*

Central catheter, n (%)	90 (74)	138 (84)	56 (84)	62 (90)	0.03

Thrombus, n (%)	2 (2)	3 (2)	3 (4)	6 (9)	*

NAITP, n (%)	1 (1)	5 (3)	5 (8)	3 (4)	*

Polycythemia, n (%)	6 (5)	12 (7)	3 (5)	5 (7)	*

RHD, n (%)	7 (6)	13 (8)	8 (12)	12 (17)	0.05

Exchange transfusion, n (%)	1 (1)	4 (2)	2 (3)	7 (10)	*

Chromosomal disorders, n (%)	0 (0)	2 (1)	1 (1)	4 (6)	*

* = No analysis possible, because some of the subgroups were too small.

GA = gestational age, SGA = small for gestational age, NEC = necrotizing enterocolitis, FFP = fresh frozen plasma, NAITP = Neonatal alloimmune thrombocytopenia, RHD = Rhesus Hemolytic Disease.

In 66% of the 105 thrombocytopenic neonates with sepsis, the low platelet count was already present before the child became ill, whereas in 15% thrombocytopenia developed after the onset of sepsis.

### Primary and secondary outcome in thrombocytopenic patient population

In the majority (87%) of neonates with an IVH ≥ grade 2 the hemorrhage occurred within the first 3 days of life. In 23% of the 48 neonates (11/48) with an IVH, the hemorrhage was discovered on the same day as the thrombocytopenia. In 20 (42%) neonates the hemorrhage was discovered after and in 16 (33%) before the thrombocytopenia even existed. In 1 neonate we could not trace the timing of the hemorrhage.

Among the 122 neonates who received a platelet transfusion 17 had an IVH ≥ grade 2. Two (13%) of these infants developed an IVH during thrombocytopenia despite platelet transfusions (one IVH grade 2, one IVH grade 3).

The primary and secondary outcome in the 4 subgroups of neonates with thrombocytopenia is presented in Table 3. Risk of hemorrhage was 10% (12/117), 14% (22/160), 6% (4/66) and 15% (10/68). We found no significant association between bleeding and severity of thrombocytopenia (p = 0.3). After logistic regression analysis, the severity of thrombocytopenia was not a significant risk factor for IVH.

**Table 3:** Primary and secondary outcome in the 4 subgroups of thrombocytopenic neonates.

	Mild **(100-149 × 10**^**9**^**/L)**	Moderate **(50-99 × 10**^**9**^**/L)**	Severe **(30-49 × 10**^**9**^**/L)**	Very severe **(<30 × 10**^**9**^**/l)**	P-value
		
	(N = 122)	(N = 164)	(N = 67)	(N = 69)	
**Primary Outcome**	(N = 117)	(N = 160)	(N = 66)	(N = 68)	

Number of neonates with IVH≥ grade 2, n (%)	12 (10)	22 (14)	4 (6)	10 (15)	0.32

IVH, grade 2	3 (3)	7 (4)	3 (5)	6 (9)	0.26

IVH, grade 3	4 (3)	6 (4)	1 (2)	2 (3)	0.86

PVHI (i.e., IVH grade 4)	5 (4)	9 (6)	0 (0)	2 (3)	0.25

**Secondary Outcome**	(N = 122)	(N = 164)	(N = 67)	(N = 69)	

Number of platelet Txs per transfused patient, median (range), N	0 (N = 0)	1 (1-1) (N = 9)	1 (1-5) (N = 48)	3 (1-28) (N = 65)	< 0.01

Mortality, n (%)	8 (7)	17 (10)	7 (10)	7 (10)	0.69

IVH = intraventricular hemorrhage, PVHI = periventricular hemorrhagic infarction, Txs = transfusions.

Logistic regression analysis showed no significant relation between mortality and the severity of thrombocytopenia (p = 0.7). In 5 patients treatment was withdrawn because of poor neurological prognosis due to major hemorrhage (IVH grade 3 or PVHI) in combination with respiratory and/or cardiac insufficiency.

Of all included neonates with thrombocytopenia 29% (122/422) received a platelet transfusion (Table 3). The median number of platelet transfusions in the mild, moderate, severe and very severe groups was 0, 1, 1 and 3, respectively. The 9 neonates in the moderate thrombocytopenia group (50-99 × 10^9^/L) were transfused because of a rapid drop in platelet count in combination with sepsis (n = 4), IVH ≥grade 2 (n = 3) or adrenal hemorrhage (n = 1). In most cases the infants with severe thrombocytopenia (30-49 × 10^9^/L) received a platelet transfusion before or during an intervention (such as a lumbar puncture or exchange transfusion), because of active bleeding or if they were clinically unstable. The rest of the newborns were transfused when the platelet count was below 30 × 10^9^/L. Only 4 neonates with very severe thrombocytopenia did not receive a platelet transfusion. Three of them had a gestational age of 37 and the fourth one of 31 weeks. All four were clinically stable, 2 had polycythemia and 2 had Rhesus hemolytic disease. None of them had IVH.

## Discussion

This study shows that although thrombocytopenic neonates are a high risk group (more unstable, and sicker than non-thrombocytopenic neonates), the severity of thrombocytopenia is not related to IVH or mortality. In 33% of the thrombocytopenic neonates with IVH grade 2 or more, IVH occurred before the thrombocytopenia even existed. Our data confirm that risk of IVH in neonates is a complex mechanism related to a wide variety of factors, of which low platelet counts is only one.

### Prevalence of thrombocytopenia

The overall prevalence of thrombocytopenia found in this study (27%) is in accordance with the rates reported in the literature for tertiary care centers (22-35%)[1-6]. The prevalence of severe (<50 × 10^9^/L) thrombocytopenia (8%) was also similar to other studies (2-25%)[1,3].

Our findings confirm that thrombocytopenia in neonates is associated with a wide variety of factors, including prematurity and low birth weight, small for gestational age, sepsis, hypotension, necrotizing enterocolitis, asphyxia, thrombi and exchange transfusions[1,4,14,21].

### Platelet transfusion

We found a positive correlation between the severity of thrombocytopenia, the duration of thrombocytopenia and with the total number of platelet transfusions. In the subgroup of infants with severe thrombocytopenia, platelet transfusion resulted in a good, but less sustained rise in platelet count (data not shown). A few studies have suggested that a fast drop in platelet count after transfusion is caused by ongoing platelet consumption instead of platelet underproduction[21].

The reason for platelet transfusions in more than half of the cases was an existing thrombocytopenia. Despite the platelet transfusions, 13% (2/17) developed an IVH (≥ grade 2), independent of the severity of thrombocytopenia. In newborns that required more transfusions (data not shown) no increased risk for IVH was seen, comparable to other studies[9,16,21]. Therefore the jury is still out on the protective value of platelet transfusions[1,4,6,9,22].

### Risk of hemorrhage

We found no significant relationship between hemorrhage and severity of thrombocytopenia. This suggests that bleeding in neonates depends on more variables than a platelet count alone. In approximately one-third of the thrombocytopenic neonates the IVH was discovered before the thrombocytopenia existed and this raises the question whether IVH can be explained as a cause or an effect of thrombocytopenia[22-24]. Hemorrhage is probably due to pre-existing fragility in vessel wall structure (especially in premature neonates) and damaged blood vessels, amongst others by cytokines and/or a co-existing coagulopathy[9,22].

Our results are important in the discussion whether thrombocytopenia is one of the major causes of IVH in neonates. Major IVH has a large impact on neurological development and mortality. However, the risk for an IVH cannot be predicted based on a platelet count alone. We also looked at other bleedings of importance in the thrombocytopenic neonates, such as pulmonary- or gastrointestinal bleedings, but the number of these hemorrhages was too small to analyze. Other variables such as gestational age, birth weight and underlying illness are of equal importance and should be taken into account. Several studies have searched for other factors that may influence the development of hemorrhage in thrombocytopenic neonates. Deficiencies, immaturity or increased consumption of other blood products, such as thrombopoietin, coagulation factors, megakaryocyte progenitor cells, cytokines and mean platelet volume, have been reported[3,25].

One of the current major issues in transfusion medicine is the appropriate trigger for platelet transfusion. Different triggers are being used for platelet transfusion. While some centers transfuse all neonates with platelet counts below 50 × 10^9^/L, other centers use lower thresholds such as < 30 or < 20 × 10^9^/L[1,2,14,17,26]. There is an apparent evolvement amongst neonatologists towards more liberal platelet transfusion practices, even in the absence of evidence based data[27]. This study does not demonstrate a difference in IVH between neonates with a platelet count below 50 × 10^9^/L or below 30 × 10^9^/L (nor between <150, <100 or lower for that matter), questioning the different transfusion thresholds. Whether platelet transfusions have a protective value in neonates with a platelet count below 20 × 10^9^/L is still not known.

## Conclusion

In this study, we found no relationship between the severity of thrombocytopenia and IVH, suggesting that the etiology of IVH in neonates is a complex multifactorial process. However, our findings should be interpreted with care due to limitations associated with the retrospective nature of the study and the relatively small sample sizes of some variables in the subgroups.

Prospective studies should be conducted to assess the true risk of hemorrhage depending on underlying conditions. Randomized controlled trials are urgently needed to determine a safe lower threshold for platelet transfusions.

## Competing interests

The authors declare that they have no competing interests.

## Authors' contributions

JSvL and TvdB made substantial contributions to the study design, data retrieval and analysis and interpretation of the data. Both were involved in writing and revising the manuscript.

EL and FJW made substantial contributions to the acquisition of data, interpretation of the data and critical revision of the manuscript. All authors have given approval of the final document.

## Pre-publication history

The pre-publication history for this paper can be accessed here:

http://www.biomedcentral.com/1471-2431/11/16/prepub
